# Efficacy of sodium-glucose co-transporter 2 inhibitors in patients with type II diabetes: A protocol for systematic review of randomised controlled clinical trials utilising a generalised pairwise modelling methodology

**DOI:** 10.1097/MD.0000000000018198

**Published:** 2019-12-20

**Authors:** Mohammed Ibn-Mas’ud Danjuma, Shaikha Al Shokri, Arwa Ibrahim YA Al Saud, Mohamed Nabil Abdelsalam Elshafei, Haajra Fatima, Suhail Doi, Mubarak Ariyo Bidmos

**Affiliations:** a Hamad General Hospital, Hamad Medical Corporation; b Weill Cornell Medicine-Qatar; c Department of Population Medicine, Qatar University Health (QU Health); d College of Medicine, Qatar University Health (QU Health), Doha, Qatar.

**Keywords:** network meta-analysis, sodium-glucose co-transporter 2 inhibitors, type II diabetes

## Abstract

**Background:**

Recent systematic reviews have evaluated the efficacy of sodium-glucose co-transporter 2 inhibitors (SGLT2) inhibitors (SGLT2I) in improving glycaemic control and mortality in patients with type II diabetes mellitus. None have incorporated the most recent study or utilized the generalized pairwise modeling methodology network meta-analysis (NMA), as well as a novel bias risk assessment approach.

**Methods:**

We propose to conduct literature search of all randomized controlled clinical trials published in English language evaluating the efficacy of (SGLT2I) versus placebo or usual standard of care from the inception of following databases to September 30, 2019: Controlled Clinical Trials Cochrane Controlled Trials Register (CCTR), Cochrane Database of Systematic Reviews (CDSR), EMBASE, Database of Abstracts of Reviews of Effectiveness (DARE), PubMed. Two reviewers will independently search these databases to identify studies that satisfy pre-specified eligibility criteria. Study bias risk assessment amongst other methodology quality evaluation of the studies will be carried out using a novel risk bias assessment tool.

**Results:**

We anticipate that the result of this review will provide additional insight into the ranking of the efficacy of various (SGLT2I) in type II diabetic patients especially as it relates to mortality, glycemic control, and body weight reduction.

**Conclusion:**

The result of this review will be useful informing therapeutic decisions by policy makers with regards to commissioning of diabetic care.

**Prospero registration number:** CRD42019139708

## 1 Introduction and rationale

There has recently been a growing interest in the sodium-glucose co-transporter 2 (SGLT2) inhibitors, a class of drugs used in the management of patients with type II diabetes.^[1]^ This has derived largely because of their proven efficacy in increasing survival,^[2,3]^ as well as reduction of body weight,^[2,3]^ and risk of heart failure^[4]^ in these cohorts of patients. With their incorporation into national and international clinical therapeutic guidelines,^[1]^ further attention has shifted to identifying the mechanisms that underlie these effects, such reduced mortality amongst others. It is well established that improving glycaemic control result in improved cardiovascular outcomes, and indeed worsens CV morbidities and mortality in certain patient populations.^[5,6]^ Furthermore, it remains a subject of ongoing debate as to the comparative efficacy of various drugs within the SGLT2 family in reducing hard cardiovascular endpoints including heart failure.^[7]^ Additionally, despite accumulating evidence pointing to their association with risk of genitourinary tract infections, the magnitude of association with each individual drug remains unknown. With the publication of recent landmark SGLT2 inhibitor trials,^[2,3,7]^ and their ongoing health technology assessments, it has become manifestly unethical to attempt head-to-head clinical trial comparison between individual drugs within the SGLT2 inhibitor family. Furthermore, newer agents within the SGLT2 inhibitor family such as Ertugliflozin,^[8]^ Tofogliflozin,^[9]^ Luseogliflozin,^[10]^ Ipragliflozin,^[11]^ and Sotagliflozin^[12]^ amongst others have obtained the requisite market authorization and entered the therapeutic scene lately. Hence there is a growing need for a robust systematic comparison of these agents utilizing publicly available data through network meta-analysis to ascertain both their hierarchy of effectiveness (HBA1c reduction, weight reduction, mortality), as well as safety considerations (renal safety, incidence of genitourinary infections). Recent systematic reviews and meta-analyses have examined efficacy and safety around the clinical utility of these drugs, but none has included most recently published studies. Furthermore, all previously published reviews^[13,14]^ have carried out comparative analyses of constituent studies using either Bayesian or frequentist NMA statistical methodologies. The exhaustive methodological flaws with these latter 2 approaches (Bayesian and Multivariate) have extensively been discussed elsewhere.^[15]^ These include numerous statistical assumptions inherent in by the 2 approaches (multivariate and Bayesian) with the demonstrable risk of compromising internal validity of error estimation, and thus potential inferences that could be drawn from the studies and interventions they seek to compare. In this review, we are utilizing the novel generalized pairwise modeling (GPM) methodology with the view to conducting direct and indirect comparisons of the effectiveness and safety of SGLT2 inhibitors through comprehensive analysis of the most up to date published reports in this area. The novelty of our approach (GPM) is the reproducibility of the same result obtained by the earlier described methods (Multivariate and Bayesian) with little or no assumptions made (except assumption for transitivity and underlying model for direct treatment effects meta-analysis).^[15]^

## 2 Objective

The key objective of this study is to carry out a systematic review with a subsequent network meta-analysis of randomized controlled trials with the view of comparing the efficacy (HBA1c, survival benefit, and weight reduction) of different SGLT2 inhibitors in patients with type II diabetes. We choose the network meta-analytic approach because it lends itself to inclusion of multiple interventions from both direct and indirect comparisons that have not been examined in comparative head-to-head clinical trial settings.

## 3 Overview

This study will be conducted in line with the recommendation of Preferred Reporting Items for Systematic Reviews and Meta-Analyses guidelines^[3]^ for meta-analyses of healthcare interventions. Additionally, the current protocol outline adheres strictly to the Preferred Reporting Items for Systematic Reviews and Meta-Analyses Protocols (PRISMA-P).^[4]^ This protocol is registered in International Prospective Register of Systematic Reviews (registration number: **CRD42019139708**).

## 4 Methodology

### 4.1 Population

Studies reporting on randomized controlled comparison between any of the SGLT2 inhibitors versus placebo or usual standard of care in patients with type II diabetes mellitus.

### 4.2 Intervention

We will accept all randomized controlled trials that evaluated any of the SGLT2 inhibitors versus placebo or usual standard of care. Usual standard of care will include all other anti-glycaemic drug classes or regimen other than SGLT2 inhibitors.

### 4.3 Study outcomes

We will accept and include all studies with atleast one of the following outcomes; the primary outcome is the mean change from baseline in HBA1c levels. The Secondary outcome includes the mean change in body weight at follow-up.

### 4.4 Study eligibility criteria

#### 4.4.1 Inclusion criteria

In this review, we will include studies that meet the following inclusion criteria:

- Age of patients ≥18 years of age.
- Randomized controlled trials only.
- Cohort comprised of type II diabetic patients only.
- Patients on at least one SGLT2 inhibitor intervention group (we have no restriction on the type of SGLT2 inhibitor regardless of the date of award of marketing authorization).
- Studies with at least one efficacy (HBA1c, mortality, weight reduction) or safety (eGFR, genitourinary infections) outcome.
- Studies published in English language up to May 31st, 2019.
- Studies that fulfil in-house rigorous in-house quality control assessment (See Appendix 1).
- Studies containing data on the selected doses for individual drugs (Empagliflozin 10 mg, Dapagliflozin 10 mg, Canagliflozin 100 mg).
- Studies with the duration of between 16 and 52 weeks

### 4.5 Exclusion criteria

All other studies that fail to meet the afore-mentioned inclusion criteria (type II diabetic patient population in randomized comparison of SGLT2 inhibitor amongst others).

### 4.6 Literature sources

Relevant studies for this review will be sources from the following publicly available databases

- Controlled Clinical Trials
- Cochrane Controlled Trials Register (CCTR)
- Cochrane Database of Systematic Reviews (CDSR)
- EMBASE
- Database of Abstracts of Reviews of Effectiveness (DARE)
- MEDLINE
- Health Technology Assessment Database (HTA)
- Index to Scientific and Technical proceedings (ISTP)
- National Research Register (NRR)
- NHS Economic Evaluation Database (NHS EED)
- Science Citation Index (SCI)
- In addition, other websites that sustain evidence-based resources would be explored. These include:
- National Coordinating Centre for Health Technology Assessment
- Scottish Intercollegiate Guidelines Network (SIGN) Guidelines
- National Institute for Clinical Excellence (NICE) (published appraisals)
- National Guideline Clearing house

### 4.7 Search strategy

This study's search strategy will aim to identify published randomized controlled clinical trials between January 1, 2000 and May 31, 2019 using the following medical search operators (Empagliflozin[tiab] OR Canagliflozin[tiab] OR Dapagliflozin[tiab] OR Sotagliflozin[tiab] OR Ertugliflozin[tiab] OR Tofogliflozin [tiab] OR ipragliflozin [tiab]) AND (Clinical Trial[ptyp] AND “humans”[MeSH Terms]) from the following sources:

- Controlled Clinical Trials
- Cochrane Controlled Trials Register (CCTR)
- Cochrane Database of Systematic Reviews (CDSR)
- EMBASE
- Database of Abstracts of Reviews of Effectiveness (DARE)
- MEDLINE
- Health Technology Assessment Database (HTA)
- Index to Scientific and Technical proceedings (ISTP)
- National Research Register (NRR)
- NHS Economic Evaluation Database (NHS EED)
- Science Citation Index (SCI)
- In addition, other websites that sustain evidence-based resources would be explored. These include:
- National Coordinating Centre for Health Technology Assessment
- Scottish Intercollegiate Guidelines Network (SIGN) Guidelines
- National Institute for Clinical Excellence (NICE) (published appraisals)
- National Guideline Clearing house

### 4.8 Criteria for study selection

All studies abstracted from publicly available electronic databases will be imported into endnote library (version X9). These studies will be “cleaned” by removing duplicates using the find “find duplicates” endnote operator. Two reviewers will independently printout the PDF version of all selected studies and store them in a separate data base, the study cohort so selected will be divided into 2 with each reviewer going through each study to ascertain each study satisfy the eligibility criteria, where disagreement exists, a third reviewer will be asked to review and adjudicate appropriately. At the end of study selection exercise, there will be a conference of all studies so evaluated for inclusion where a harmonized and an agreed list of included studied will be arrived at.

## 5 Interventions

Studies included in this review are those for which there is at-least one randomized controlled evaluation of one SGLT2 inhibitor, with either a placebo or the usual standard of care.

For the purpose of this review, studies of less than 16 weeks and greater than 52 weeks will be excluded.

Apart from primary trial interventions, studies that failed to report robustly how they dealt with rescue treatment such as admission for hypoglycaemic episodes will be excluded.

### 5.1 Data collection/abstraction

Two reviewers will develop and pilot an excel data collection database on 3 randomly selected studies. This form will be used by individual reviewers (MAB, MID, AES) to extract data from selected studies. Variables of interest includes Study ID, year of publication, duration of follow up, study first author, active comparator, control comparator (placebo/usual standard of care), number of patients and effect sizes of both primary and secondary outcome, that is, standard error (SE), standard deviation (SD), and confidence interval (CI).

In cases where the standard error is not provided, we will abstract the upper and lower range of confidence interval in order to calculate the SE. All studies will be identified within the Microsoft excel specific database by the first author's last name and the year of publication.

### 5.2 Study bias risk assessment

We choose a novel framework for risk bias assessment recently published by Stone et al^[16]^ to assesses the potential risk bias of the methodological quality of the RCT's we selected for this review. This checklist, which consists of 36 standard questions, ascertains study bias risk assessment across 9 domains (see appendix 1 for the comprehensive check list). The novelty of this approach is its ability to mitigate for methodological flaws across study designs and other factors that may compromise the internal validity of our study. Additionally, the check list proposed by this approach^[16]^ incorporates and unifies previous approaches (including the Cochrane check list^[17]^). In the risk bias assessment of the studies selected for this review, we will consider the mode of randomization as satisfactory if the mode of concealment includes computer-based programs, sequence randomization, or sealed envelopes were used. We will assess for the blinding of study investigators, participants, and analysts to various allocation groups. We will adjudge blinding as satisfactory if the afore-mentioned groups are completely blinded to various randomized group assignment. Additionally, we will classify attempts at reducing information bias as adequate if amongst others, care was delivered equally to both groups, co-interventions that could impact the outcome were comparable between groups or avoided, and if study outcome criteria were consistently applied. The other standards, domains, and safeguards within this novel check list is given in appendix 1. Each reviewer (MID, MAB) will score individual studies against the 36 safeguards, domains, and questions on the check list with a summary score provided for each study. We will consider scores within all the 9 domains in determining the overall risk bias quality of each study. Amongst the 9 domains, we will report on the most important source of bias. Where disagreement arose from scoring of studies between the 2 reviewers, this will be resolved initially by a conference between the reviewers or by the third reviewer (AES).

### 5.3 Data analyses/synthesis

#### 5.3.1 Estimation/calculation of effect sizes

All analyses will be conducted using the natural log of odds ratio (OR) and then transformed back to OR for presentation purposes. For the primary outcome (HBA1c) all initial analyses will be carried out using the log of mean change from baseline and subsequently transformed back to mean change from baseline. Where this is not reported, it will be calculated from data/coefficients reported in the study. If data are not available to calculate OR, it will be requested from the study authors (including the relevant confidence intervals). Where study authors are unable or unwilling to provide requested missing data, we will determine the missing data from coefficient provided, for example, from CI as well as relevant means at baseline or at follow-ups.

Secondary outcomes will be calculated using the same procedure as for primary outcome. If a study includes both direct and indirect comparisons, only direct comparison data will be included given that the primary focus of the present study is to compare the efficacy outcomes between different SGLT2 inhibitors.

#### 5.3.2 Pooled estimates for change in outcomes

Network maps will be drawn to illustrate the interventions that are directly compared against each other, including the amount of evidence available for each treatment and its comparator. Additionally, we will draw a separate network maps for each of our study outcome. The most dominant comparison for each network estimate will be determined by constructing individual contribution plots for each outcome.

#### 5.3.3 Meta-biases evaluation

Other potential publication bias including small-study effects will be evaluated using comparison-adjusted funnel plots. The adjustment is necessary as it accounts for the fact that funnel plots in network meta-analysis estimate actual treatment effects (in contrast to what obtains in meta-analysis). It therefore meant that no single reference line from which symmetry can be evaluated.

Statistical analysis and analytical software for data synthesis

### 5.4 Analytical software for data synthesis

All statistical analysis will be carried out with MetaXL software (version 5.3 © EpiGear International Pty Ltd ABN 51 134 897 411 Sunrise Beach, Queensland, Australia, 2011–2016).

## 6 Statistical analysis

Data for statistical analyses as alluded to in earlier in sections will be extracted into a Microsoft Excel file. Our primary outcome is a continuous variable; therefore, we will calculate the effect sizes of the effect of active versus control interventions on HBA1c using the weighted mean difference (WMD). For trials that fail to provide adjusted mean change of HBA1c at the end of follow-up, this will be calculated using the mean HBA1c at baseline and at the end of follow-up as appropriate. We will then calculate the 95% CI for each single WMD, and the results will be pooled using the random or fixed effect models (depending on the value of *Q*-statistics/*I*^2^).

### 6.1 Strategy for dealing with missing data

We anticipate that we will encounter missing data in the course of analyses of the studies we will include in this review. Our strategy for dealing with this involves initially liaising with the corresponding authors of the studies involved for any additional input on missing data/parameters. Where study authors are unable or unwilling to provide requested missing data, we will determine the missing data from coefficients provided for example from confidence intervals as well as relevant means at baseline and at follow up. Additionally, we will also attempt imputation of random values as appropriate to ensure validity of this strategy we will do an overall meta regression analysis of these estimates.

### 6.2 Sensitivity analysis

We will carry out a sensitivity analysis to ascertain if the combined estimates of the treatments are dominated by one or group of several studies. More so if these studies have poor risk scores from our bias check list (high risk of bias). These studies will be excluded from analyses to determine their robustness of estimates with and without them. Additionally, we will determine if imputation of missing values or utility of different coefficients (SE and 95% CI) have any bearing on study results, and if it does, which is better.

### 6.3 Ascertainment of heterogeneity

We will estimate both *Q*-statistics and *I*^2^ to estimate the degree and magnitude of heterogeneity (between each pairwise comparison) amongst our selected studies.

### 6.4 Ascertainment of transitivity and similarity

Transitivity and similarity pose enormous challenge when attempts are made to define them statistically. As in both the multivariate and Bayesian approaches, utilizing our GPM methodology, we will assume that the adjustment of the indirect comparison between treatments will be based on transitivity and the fidelity of our meta analytical methods.^[15]^

### 6.5 Assessment of inconsistency

Owing to the multiple nature of the loops we expect in our network meta-analysis, we will employ H^2^ derived from Cochrane *Q*-statistics to directly ascertain the consistency or otherwise of our estimates.^[15]^ For each network result estimate, we will compute the *H*^2^ value (or its square root, *H*), and this will then serve as the inconsistency per comparison made. Ultimately, for the final and the overall network, we will then compute the weighted average *H*^2^ or *H* over all final comparisons, and we will then report this as a measure of inconsistency across the entire network.^[15]^

## 7 Discussion

Through our GPM methodology of network meta-analysis, we hope to have a more robust outline of the most uptodate ranking of individual members of the SGLT2 inhibitor class regarding their efficacy in patients with type II diabetes. This will be very useful in providing guidance for guideline updates and potentially inform decision making in diabetic medicines and therapeutic committees in various healthcare delivery settings. Additionally, our result may provide insight towards generating hypothesis for development of future prospective studies in this area.

## Acknowledgment

The publication of this article was funded by the Qatar National Library.

## Author contributions

**Conceptualization:** Mohammed Ibn-Mas’ud Danjuma, Suhail Doi, Mubarak Ariyo Bidmos.

**Data curation:** Mohammed Ibn-Mas’ud Danjuma, Shaikha Al Shokri, Arwa Ebrahim Al Saud, Mohamed Nabil Abdelsalam Elshafei, Haajra Fatima, Mubarak Ariyo Bidmos.

**Formal analysis:** Mohammed Ibn-Mas’ud Danjuma, Shaikha Al Shokri, Mohamed Nabil Abdelsalam Elshafei, Suhail Doi, Mubarak Ariyo Bidmos.

**Investigation:** Mohammed Ibn-Mas’ud Danjuma, Shaikha Al Shokri.

**Methodology:** Mohammed Ibn-Mas’ud Danjuma, Shaikha Al Shokri, Arwa Ebrahim Al Saud, Mohamed Nabil Abdelsalam Elshafei, Haajra Fatima, Mubarak Ariyo Bidmos.

**Project administration:** Mohammed Ibn-Mas’ud Danjuma, Mohamed Nabil Abdelsalam Elshafei, Haajra Fatima.

**Resources:** Arwa Ebrahim Al Saud.

**Validation:** Arwa Ebrahim Al Saud, Mohamed Nabil Abdelsalam Elshafei.

**Writing – original draft:** Mohammed Ibn-Mas’ud Danjuma, Arwa Ebrahim Al Saud, Mubarak Ariyo Bidmos.

**Writing – review & editing:** Mohammed Ibn-Mas’ud Danjuma, Shaikha Al Shokri, Arwa Ebrahim Al Saud, Haajra Fatima, Suhail Doi, Mubarak Ariyo Bidmos.

Mohammed Ibn-Mas’ud Danjuma orcid: 0000-0003-2198-5278.

Mubarak Bidmos orcid: 0000-0002-4977-3421

Suhail Doi orcid: 0000-0002-2630-2125

Haajra Fatima orcid: 0000-0002-5535-3140
